# Risk factors for arterial catheter failure and complications during critical care hospitalisation: a secondary analysis of a multisite, randomised trial

**DOI:** 10.1186/s40560-024-00719-1

**Published:** 2024-03-08

**Authors:** Jessica A. Schults, Emily R. Young, Nicole Marsh, Emily Larsen, Amanda Corley, Robert S. Ware, Marghie Murgo, Evan Alexandrou, Matthew McGrail, John Gowardman, Karina R. Charles, Adrian Regli, Hideto Yasuda, Claire M. Rickard, the RSVP Study Investigators

**Affiliations:** 1 Metro North Health, Herston Infectious Disease Institute, Herston, Queensland Australia; 2 School of Nursing, Midwifery and Social Work, The University of Queensland, Brisbane, Queensland Australia; 3 Alliance for Vascular Access Teaching and Research Group, Nathan, Queensland Australia; 4 Nursing and Midwifery Research Centre, Royal Brisbane and Women’s Hospital, Herston, Queensland Australia; 5 School of Medicine and Dentistry, Griffith University, Brisbane, Queensland Australia; 6 Menzies Health Institute Queensland, Griffith University, Brisbane, Queensland Australia; 7 School of Nursing and Midwifery, Griffith University, Brisbane, Queensland Australia; 8 School of Nursing, University of Wollongong, Wollongong, New South Wales Australia; 9 Department of Intensive Care, Liverpool Hospital, Liverpool, New South Wales Australia; 10 Rural Clinical School, The University of Queensland, Toowoomba, Queensland Australia; 11 Intensive Care Service, Royal Brisbane and Women’s Hospital, Herston, Queensland Australia; 12 School of Medicine, The University of Queensland, Herston, Queensland Australia; 13 Department of Intensive Care, Fiona Stanley Hospital, Perth, Western Australia Australia; 14 Medical School, The Notre Dame University, Fremantle, Western Australia Australia; 15 Medical School, The University of Western Australia, Perth, Western Australia Australia; 16 Department of Emergency and Critical Care Medicine, Jichi Medical University Saimata Medical Center, Saitama, Japan; 17 Department of Clinical Research Education and Training Unit, Keio University Hospital Clinical and Translational Research Center, Tokyo, Japan

**Keywords:** Arterial catheter, Secondary analysis, Complication, Healthcare associated infection, Intensive care

## Abstract

**Objectives:**

Arterial catheters (ACs) are critical for haemodynamic monitoring and blood sampling but are prone to complications. We investigated the incidence and risk factors of AC failure.

**Methods:**

Secondary analysis of a multi-centre randomised controlled trial (ACTRN 12610000505000). Analysis included a subset of adult intensive care unit patients with an AC. The primary outcome was all-cause device failure. Secondary outcomes were catheter associated bloodstream infection (CABSI), suspected CABSI, occlusion, thrombosis, accidental removal, pain, and line fracture. Risk factors associated with AC failure were investigated using Cox proportional hazards and competing-risk models.

**Results:**

Of 664 patients, 173 (26%) experienced AC failure (incidence rate [IR] 37/1000 catheter days). Suspected CABSI was the most common failure type (11%; IR 15.3/1000 catheter days), followed by occlusion (8%; IR 11.9/1,000 catheter days), and accidental removal (4%; IR 5.5/1000 catheter days). CABSI occurred in 16 (2%) patients. All-cause failure and occlusion were reduced with ultrasound-assisted insertion (failure: adjusted hazard ratio [HR] 0.43, 95% CI 0.25, 0.76; occlusion: sub-HR 0.11, 95% CI 0.03, 0.43). Increased age was associated with less AC failure (60–74 years HR 0.63, 95% CI 0.44 to 0.89; 75 + years HR 0.36, 95% CI 0.20, 0.64; referent 15–59 years). Females experienced more occlusion (adjusted sub-HR 2.53, 95% CI 1.49, 4.29), while patients with diabetes had less (SHR 0.15, 95% CI 0.04, 0.63). Suspected CABSI was associated with an abnormal insertion site appearance (SHR 2.71, 95% CI 1.48, 4.99).

**Conclusions:**

AC failure is common with ultrasound-guided insertion associated with lower failure rates.

*Trial registration* Australian New Zealand Clinical Trial Registry (ACTRN 12610000505000); date registered: 18 June 2010.

**Supplementary Information:**

The online version contains supplementary material available at 10.1186/s40560-024-00719-1.

## Introduction

Arterial catheterisation is a common procedure in intensive care and anaesthetic departments worldwide. More than 10 million arterial catheters (ACs) are placed in the United States and Europe each year [1–3] to support continuous haemodynamic monitoring, blood sampling [2, 4] or arterial blood gas monitoring. ACs are associated with risks such as infection [2, 5–8], occlusion [4, 9–12], thrombosis [5, 6, 9, 13–16], and dislodgement [16, 17]. Such complications contribute to substantial patient morbidity by prolonging intensive care unit (ICU) length of stay [1, 14, 18], thereby increasing potential health care costs.

International cohort studies suggest significant variation in AC use and maintenance practices—more so than with central venous catheters [19–21]. This variation likely stems from a lack of data regarding appropriate AC maintenance and risk factors associated with AC complications [19, 20]. Few studies have investigated complications using multivariable analysis techniques, and all have focussed solely on infection outcomes [22–27]. A detailed understanding of such risk factors, and conversely, the protective factors is lacking. To address this gap, we conducted a secondary analysis of a multi-site randomised controlled trial (RCT) which compared the effectiveness and costs of 7-day (intervention) versus 4-day (control) infusion set replacement in patients requiring central venous and peripheral arterial access. Our objectives were to: i) determine the prevalence and cause of AC failure and device complications; and ii) determine predictors of AC failure and device complications. We hypothesised that specific patient-, provider- (inserter) and catheter-related characteristics would be associated with AC failure and complications. Given the omnipresence of ACs in the ICU we sought to identify modifiable risk factors which may inform interventions for future clinical trials.

## Methods

### Study design and sample population

We conducted a secondary analysis of data from a multi-site RCT. All adult patients aged > 16 years with an AC were included. Ethical approval was obtained from Griffith University (Ref No: 2021/834). The study reporting follows the Strengthening the Reporting of Observational Studies in Epidemiology (STROBE) guidelines [28].

The parent study, from which the data was sourced, is the Replacement after Standard Versus Prolonged use (RSVP) trial, which tested the effect of 4- versus 7-day infusion set replacement intervals [17] in a ten-site (including 5 ICUs; all level 3 facilities) Australian RCT conducted between May 2011 and December 2016 [17] (ACTRN12610000505000). RSVP enrolled 2944 patients of all ages (excluding neonatal ICU patients). Eligible patients required the insertion of a central venous and/or AC, with the device in situ for > 24 h, and expected to be used for ≥ 7 days [29]. The primary endpoint was catheter-related bloodstream infections. Sites obtained institutional review approvals and informed written consent was obtained or waived as per local ethical requirements. Of the 2944 patients enrolled in RSVP, 664 adults received a PAC.

### Outcomes

The primary outcome was all-cause AC failure, defined as cessation of catheter function prior to the completion of necessary therapy [30]. Secondary outcomes were individual complications including: suspected catheter associated bloodstream infection ([CABSI]; provider assessed) or microbiologically proven CABSI, defined in line with international recommendations [31], AC occlusion, thrombosis, accidental removal, or line fracture [32, 33].

### Variables

The parent study (RSVP) collected patient demographic and clinical characteristics including age, sex, admission diagnosis, ICU length of stay, ventilation requirement and severity of critical illness (The Acute Physiology and Chronic Health Evaluation [APACHE II]), as well as device and provider characteristics (e.g., insertion site, catheter material, insertion technique and inserter designation) for each participant. Data were collected using the web-based platform Research Electronic Data Capture (REDCap; Vanderbilt University) [34].

### Statistical analysis

Participant demographic and AC characteristics (1 AC per participant was studied) are reported descriptively using frequency (percentage) for categorical data and mean (standard deviation) or median (interquartile range; IQR) for continuous variables depending on normality of distribution. The incidence of device failure was calculated using Poisson regression, offset by the natural logarithm of days at risk. Failure and complications are presented as incidence rate per 1000 catheter days with 95% confidence interval (CI). To investigate risk factors for failure, a Cox proportional hazards model was used for all-cause failure. When investigating the component failure outcomes suspected/confirmed CABSI, blockage and accidental removal, competing-risks regression models were used to account for possible failure due to other reasons. Multivariable models were not constructed for other failure types due to low incidence. Risk estimates are presented as hazard ratio (HR) for failure, and sub-hazard ratio (SHR) for complication types. Patient, clinical and device characteristics, but not the RCT study group, were considered for inclusion in the best multivariable model. The Bayesian information criterion (BIC) statistic was used to identify the model with the most explanatory power relative to its complexity. The BIC was calculated for all possible models and the model with the smallest BIC was chosen as the best final model. Kaplan Meier and competing-risks regression curves were plotted. All analyses were performed using Stata v15.1 (StataCorp, College Station, TX).

## Results

### Patient characteristics

**Fig. 1:**
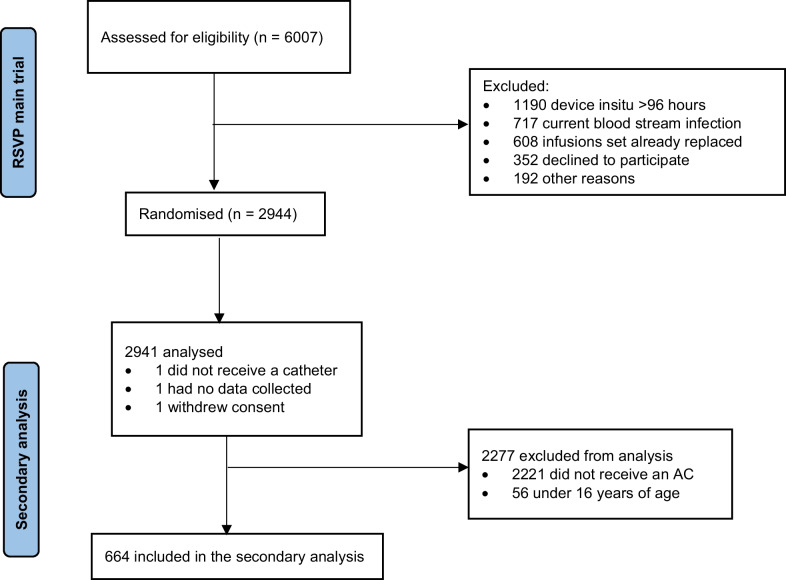
Patient flow chart. Uploaded as high quality DPI image

Included in this secondary analysis were 664 adults who required an AC (Fig. 1). Patients were predominately male (*n* = 449; 68%), aged between 16 and 59 years (*n* = 346; 52%) and admitted for a medical condition (n = 316, 48%; Table 1). Eighteen percent (*n* = 118) of patients had a current infection, most commonly respiratory (*n* = 65; 10%). Seventy-nine percent (*n* = 524) of patients required mechanical ventilation, with the median ICU length of stay, at time of device insertion 4 days (IQR 3, 4).

**Table 1:** Participant characteristics

Category	Arterial catheters (*n* = 664; %)
Age	
16–59	346 (52)
60–74	211 (32)
75+	107 (16)
Hospital day at entry (N = 657)	
0–3	182 (28)
4–7	314 (48)
8+	161 (24)
Gender	
Male	449 (68)
Female	215 (32)
Diagnosis	
Medical	316 (48)
Surgical elect	84 (13)
Surgical cardiac	86 (13)
Surgical emergency	105 (15)
Trauma and burns	69 (10)
Other	4 (1)
ICU APACHE II (*N* = 662)	
0–9	51 (8)
10–19	309 (46)
20–29	244 (37)
30–39	53 (8)
40–49	5 (1)
Catheter—side of body	
Left	274 (41)
Right	390 (59)
Diabetes (*N* = 663)	
No	537 (81)
Yes	126 (19)
Leukopenia*	
No	643 (97)
Yes	21 (3)
Artery—AC	
Radial	551 (83)
Femoral	49 (8)
Dorsalis pedis	16 (2)
Other	48 (7)
Current infection (at entry)	
No	545 (82)
Yes	119 (18)
Infection on admission	
Respiratory	65 (10)
Wound	10 (2)
Urinary	6 (1)
Other	28 (4)
Multiple	10 (1)
Site check^+^ (*N* = 662)	
Normal	633 (95)
Red—only	24 (3)
Swelling—only	1 (1)
Multiple	4 (1)
Multiple insertion attempts (*n* = 653)	
No	638 (98)
Yes	15 (2)
Additional IVDs (at removal) (N = 17)	
0	1 (6)
1	12 (70)
2	1 (6)
3 +	3 (18)
Dressing and securement (at entry) (Total = 100%)	
Simple transparent	92 (14)
Advanced transparent	3 (1)
Multiple	569 (85)
Ultrasound guided insertion (*n* = 657)	
No	501 (76)
Yes	156 (24)
Place of insertion	
ICU	422 (64)
OT	175 (26)
Other	67 (10)
Patient ventilated	
No	140 (21)
Yes	524 (79)
Received IV antibiotics (during enrolment)	
No	114 (17)
Yes	550 (83)
Received IV heparin lock/flush (during enrolment)	
No	649 (98)
Yes	15 (2)
Received IV heparin infusion (during enrolment)	
No	610 (92)
Yes	54 (8)

^*^White blood cell count < 1.0 × 10^9/L within 72 h of trial entry; ^+^ at any time; ICU: intensive care unit; APACHE: The Acute Physiology and Chronic Health Evaluation; AC: arterial catheter; IVDs: intravenous devices; IV: intravenous

Figure 1 Flowchart illustrating how the analysis population evaluated in this secondary analysis was derived from the overall intention-to-treat population of the RSVP trial.

### AC characteristics

All ACs were inserted by physicians, predominately in ICU (*n* = 422; 64%). ACs were generally inserted on the first attempt (*n* = 638; 98%), using landmark technique (*n* = 501; 76%) in the radial artery (*n* = 551; 83%). ACs were either peripheral venous catheters or arterial catheters (with introducer), secured with multiple dressing and securement products (*n* = 569; 85%), including simple transparent dressings. Heparin saline (2 iu/ml) or 0.9% normal saline were used in pressurised transducer tubing depending on local hospital policy.

### AC and patient outcomes

Of the 664 patients with ACs, 491 (74%) completed therapy. One hundred and seventy-three patients (26%) experienced AC failure (Table 2). Median AC dwell was 6.5 days (IQR 4.8, 8.5) in all catheters and 4.8 days (IQR 3.5, 6.5) in catheters which failed. Most catheters were removed during daylight hours (0700–1900 h; *n* = 534; 80%). All-cause AC failure incidence rate (IR) was 37.0 per 1000 catheter days (95% confidence interval [CI] 31.7, 42.7). Suspected CABSI, with 11% was the most common reason for premature device removal (IR 15.3; 95% CI 12.2, 19.3). AC occlusion occurred in 8% of patients (IR 11.9, 95% CI 9.2, 15.5), followed by 4% accidental removal (IR 5.5; 95% CI 3.8, 8.1). CABSI was confirmed in 2% equating to 16/72 (22%) of suspected CABSI. Central venous catheters were ruled out as the source of the confirmed CABSI in ACs. Removal for fracture, pain and thrombus were rare.

**Table 2:** Incidence of failure (173 failures from 664 catheters)^a^

Reason	Arterial catheters (N = 664), *n* (%)	IR (95% CI)
All cause failure^b^	173 (26)	36.8 (31.7 to 42.7)
Suspected CABSI	69 (10)	14.7 (11.6 to 18.6)
Occlusion	55 (8)	11.7 (9.0 to 15.2)
Accidental removal	25 (4)	5.3 (3.6 to 7.9)
Proven CABSI	16 (2)	3.4 (2.1 to 5.6)
Fractured	4 (< 1)	0.9 (0.3 to 2.3)
Painful	3 (< 1)	0.6 (0.2 to 2.0)
Thrombus	1 (< 1)	0.2 (0.0 to 1.5)

IR: incidence rate; CI: confidence interval; CABSI: catheter associated bloodstream infection

^a^Each device could have more than one complication; ^b^Fractured, painful, occlusion, accidental removal, thrombus, suspected CABSI, CABSI

Univariable associations of patient, provider (inserter) and device characteristics with all types of AC failure are outlined in Additional file 1. The most appropriate multivariable models as identified using BIC are presented in Additional file 2.

### Risk factors for all-cause failure

Variables associated univariably with increased AC failure were: female gender (HR 1.38, 95% CI 1.01, 1.88), abnormal site check (on nursing assessment; HR 2.09, 95% CI 1.31, 3.34), surgical emergency admission (HR 2.35, 95% CI 1.59, 3.47), or trauma and burns admission (HR 2.19, 95% CI 1.43 to 3.33), compared to a medical admission. Variables associated with decreased AC failure were increasing years of age (60–74 years, HR 0.64 95% CI 0.46, 0.91;75 + years, HR 0.36, 95% CI 0.21, 0.62) compared to being aged 16–59 years, ultrasound-guided AC insertion (HR 0.45, 95% CI 0.30, 0.69), antibiotics during AC dwell (HR 0.65, 95% CI 0.45, 0.93), and higher APACHE score (APACHE 20–29, HR 0.50, 95% CI 0.29, 0.86; APACHE 30–49 HR 0.49, 95% CI 0.22, 1.00) compared with an APACHE of 10–19).

When considering multivariable models (Table 3), ultrasound insertion (HR 0.48, 95% 0.31, 0.73) and increasing age (60–74 years HR 0.63, 95% CI 0.44 to 0.89; 75 + years HR 0.36, 95% CI 0.20, 0.64) compared to age 15–59 years were the two variables included in the most parsimonious model, with both models associated with reduced AC failure risk.

**Table 3:** Association between risk factors and device failure identified by multivariable Cox Regression (*N* = 664)

Variable	All cause failure	Suspected CABSI	Occlusion
	Hazard ratio (95% CI)	Sub hazard ratio (95% CI)	Sub hazard ratio (95% CI)
Ultrasound Yes	0.48 (0.31 to 0.73)		0.12 (0.03 to 0.49)
Age			
16–59	*Reference*	^	^
60–74	0.63 (0.44 to 0.89)		
75+	0.36 (0.20 to 0.64)		
Site check			
Not normal	^	2.71 (1.48, 4.99)	
Diabetes Yes	^	^	2.44 (1.43 to 4.18)
Gender Female	^	^	0.18 (0.04 to 0.73)

No variables significantly associated with ‘Proven CABSI’ or ‘Accidental Removal’ in best BIC model

CI: confidence interval; ICU: intensive care unit; CABSI: catheter associated bloodstream infection

3 suspected CABSIs were confirmed. ^not included in multivariable model

### Risk factors for suspected CABSI

For suspected CABSI, univariable analyses identified AC placement in the femoral artery (SHR 2.21, 95% CI 1.15, 4.23) rather than the radial artery, trauma/burns diagnosis (SHR 2.61, 95% CI 1.42, 4.79) compared to medical diagnosis, and abnormal AC site on nursing check (SHR 2.70, 95% CI 1.47 to 4.96) as factors associated with increased risk. Being aged 60–74 years decreased risk compared to being aged 15–59 years (SHR 0.30 95% CI 0.11, 0.83).

On multivariable analyses, abnormal AC site on nursing check remained associated with suspected CABSI (SHR 2.71, 95% CI 1.48, 4.99).

### Risk factors for proven CABSI

No variables were identified as being significantly associated with proven CABSI.

### Risk factors for occlusion

On univariate analyses, AC occlusion was higher in females (SHR 2.66, 95% CI 1.57, 4.53), trauma or burn injuries (SHR 2.24, 95% CI 1.05, 4.80), or a surgical emergency (SHR 3.18, 95% CI 1.67, 6.02) compared to a medical admission, and in patients with AC placement in the dorsalis pedis rather than the radial artery (SHR 3.03, 95% CI 1.08, 8.45). Ultrasound-guided insertion (SHR 0.11, 95% CI 0.03, 0.47), receipt of antibiotic therapy (SHR 0.50, 95% CI 0.28, 0.90), and having a diabetes comorbidity (SHR 0.15, 95% CI 0.04, 0.63) were associated with less AC occlusion, as were increasing age (60–74 years, SHR 0.49, 95% CI 0.26, 0.93; 75 + years, SHR 0.32; 95% CI 0.11, 0.88) compared to being aged 16–59 years.

In the best multivariable model, ultrasound-guided insertion and diabetes remained associated with reduced occlusion (SHR 0.12, 95% 0.03, 0.49 and 0.18, 0.04, 0.73 respectively), whilst female gender remained associated with an increased risk of occlusion (SHR 2.44, 95% CI 1.43, 4.18; Table 3).

### Risk factors for accidental removal

On univariable analyses, accidental AC removal was more likely to occur in patients admitted to ICU following a surgical emergency, compared to admission for a medical diagnosis (SHR 3.17, 95% CI 1.25, 8.03). Patients with an APACHE of 0–9 were more likely to experience accidental removal when compared with patients with an APACHE of 10–19 (SHR 3.47, 95% 1.18, 10.20).

No factors remained significantly associated with accidental removal in the multivariable models.

## Discussion

In this heterogeneous adult ICU cohort, unplanned early removal of ACs was common (1 in 4 ACs). ACs were most commonly removed due to suspected infection, followed by occlusion and accidental removal. We showed that ultrasound-guided AC insertion and increasing patient age reduced the relative risk of catheter failure, and that females have more than double the risk of AC occlusion, following adjustment for other patient, provider, and device specific factors.

Similar to peripheral IV catheter insertion [35, 36] we showed that use of ultrasound for AC insertion was a protective factor against all cause failure, suspected infection, and occlusion [37, 38]. Despite finding a high first attempt insertion rate, data suggests ultrasound use for AC insertion is beneficial, supporting appropriate vessel and site selection, shorten insertion time, and enhance procedural accuracy [39–41]. In this cohort only 24% of ACs were inserted using ultrasound, demonstrating the device is overlooked for this procedure despite international guidelines such as The Infusion Therapy Standards of Practice [42] recommending ultrasound use for AC insertion. Previous studies offer explanations for this lack of uptake, highlighting barriers such as resourcing, training, and organisational support as key contributors to the ad hoc use of ultrasound [38, 43, 44].

Suspected CABSI was the most common reason for premature device removal and patients who had their AC inserted with ultrasound had an almost twofold-reduced risk of suspected CABSI. While clinical practice varies, catheter-related infection is typically suspected when the patient exhibits new and unexplained signs of sepsis and the catheter has been in place for more than 4 days, and suspicion is heightened by any redness or discharge from the AC site [45]. Guidelines [46] for preventing catheter-related infections recommend that catheter cultures are performed when a catheter is removed for suspected infection so as to enable diagnosis and bacterial identification. Yet AC removal often requires insertion of a replacement device, while diagnosis of bloodstream infection and causative pathogens is time-consuming and retrospective. This may explain why many suspected CABSI cases lead to AC removal without cultures being taken, a situation likely leading to underdiagnosis of confirmed CABSI. Point of care diagnostics for the rapid detection of bacteraemia in ICU remain limited in application however would facilitate fast diagnosis, timely treatment and potentially decrease the volume of ACs removed on suspicion of infection [47]. This would be particularly useful in confirming diagnosis in ICU patients who typically have a higher bacterial load, while protecting those who have poor vessel health from unnecessary removals [48].

Our findings suggest that AC occlusion is a key complication necessitating premature device removal in ICU. AC occlusion often occurs in ICU due to intimal hyperplasia, intima–media thickening and luminal thrombosis [10]. We identified patients with diabetes who were 2.4 times more likely to develop an AC occlusion. Evidence suggests this may be a result of the increased platelet responsiveness (hyperactivity), making patients with diabetes more prone to developing thromboses [49]. Further diabetes is associated with atherosclerotic narrowing of peripheral arteries and thus may contribute to an increased rate of occlusions [50]. Female gender was the strongest, non-modifiable predictor of AC occlusion. Female patients were 2.7 times more likely to experience catheter blockage compared to male counterparts. Concerningly, full recovery of radial arterial blood flow can take up to 7 days post AC removal [51]. Our finding aligns with existing evidence which demonstrates female patients are three times more likely to develop AC thrombosis [15]. An increased thrombosis risk in females has also been demonstrated in other vascular catheters including peripheral intravenous catheters [52]. Studies have attributed this increased risk to females having higher fibrin production and reduced markers of fibrinolysis, thereby increasing their overall coagulation potential [53]. Further, females typically have a smaller vessel diameter, in this case arterial, which can contribute to stenosis and occlusion if the catheter to vein ratio is consistently reduced [54, 55]. Females’ predisposition towards higher fibrin generation highlights the need to ensure correct technique for blood draws on arterial catheters. Shear rates and turbulence – the speed at which the blood draw is completed—is an important factor in the coagulation cascade and intima–media thickening, with both platelet adhesion and activation and thrombin generation increased under conditions of shear stress [56, 57]. Further work should examine the impact of interventions which reduce the risk of catheter thrombosis (e.g., AC gauge) and address women’s unique clotting factors (e.g., addressing the clotting cascade, AC material).

Our analysis is associated with several strengths. First, we used high-quality data from a large multisite RCT, prospectively collected. Secondly, device complications were prospectively monitored using rigorous definitions and, in the case of CABSI, blinded outcome assessors. The finding of increasing age being a protective factor against AC failure may be related to the reduced inflammatory response in elderly patients [58], or the effect of aging on vascular endothelium and structural integrity in arteries [59] which warrants further enquiry, Our analysis has some limitations, mainly its exploratory nature and its setting in one country. Due to the low event rates, results should be interpreted with caution, particularly the accidental removal analysis which had fewer patients. Secondly, as a secondary analysis we were not able to collect additional data restricting the number of predictor variables in risk-adjustment models points (e.g., sedation level, coagulation profile/ platelet inhibition, institutional practices, race). Third, routine replacement may have been a competing risk with 20% of devices routinely replaced, a practice not recommended in clinical guidelines. In the pragmatic RCT, AC maintenance was based on local guidelines informed by international evidence (Centre for Disease Control guidelines) [41]. There was some variation in AC maintenance and we did not adjust for site-level clustered variables such as use of heparinized or non-heparinized saline flush infusions, although these have not been shown effective in prior research [60]. Overall, further interventional studies are required to ascertain the benefit of strategies in female patients to reduce thrombosis risk and understand factors promoting translation of ultrasound guided AC insertion for patients across critical care and anaesthetic settings.

## Conclusion

Our secondary analysis of the RSVP trial demonstrated a clinically concerning incidence of AC failure. We identified younger age to be associated with increased risk, female patients as at significantly higher risk of occlusion, while patients with diabetes had reduced risk. Ultrasound-guided insertion was significantly associated with reduced AC failure and occlusion, lending support to increased use of this technology.

### Supplementary Information

**Additional file 1. **Risk factors for catheter failure and complications (univariate analyses).

**Additional file 2. **Bayesian information criterion values by failure type.

## Data Availability

The data that support the findings of this study may be made available on request to the corresponding author, but ethical restrictions apply to the availability of these data, and so are not publicly available. Data may be made available upon reasonable request and with permission of Griffith University HREC at research-ethics@griffith.edu.au.

## Abbreviations

AC: Arterial catheter
APACHE II: Acute Physiology and Chronic Health Evaluation
BIC: Bayesian Information Criterion
CABSI: Catheter associated blood stream infection
CI: Confidence Interval
HR: Hazard Ratio
ICU: Intensive care unit
IR: Incidence rate
IQR: Interquartile range
RCT: Randomised controlled trial
RSVP: Replacement after Standard Versus Prolonged use trial
SHR: Sub-hazard ratio
STROBE: Strengthening the Reporting of Observational Studies in Epidemiology
