# Supplementary material for: Risk factors for arterial catheter failure and complications during critical care hospitalisation: a secondary analysis of a multisite, randomised trial
Source: J Intensive Care. 2024 Mar 8;12:12. doi: 10.1186/s40560-024-00719-1 (PMC10924392; doi:10.1186/s40560-024-00719-1)
Supplement: Supplementary file 1 — Additional file 1. Risk factors for catheter failure and complications (univariate analyses). [file 40560_2024_719_MOESM1_ESM.docx]

**Additional file 1. Risk Factors for catheter failure and complications (univariate analyses)**

| Category | All cause failure  HR (95% CI) p-value  (n = 173; 26.1%) | Suspected CABSI  SHR (95% CI) p-value  (n = 69; 10.4%) | Proven CABSI  SHR (95% CI) p-value  (n=16; 2.4%) | Occlusion  SHR (95% CI) p-value  (n = 55; 8.3%) | Accidental removal  SHR (95% CI) p-value  (n = 25; 3.8%) |
| --- | --- | --- | --- | --- | --- |
| Age |  |  |  |  |  |
| - 16-59 | Reference | Reference | Reference | Reference | Reference |
| - 60-74 | 0.64 (0.46 to 0.91) p=0.01 | 0.79 (0.48 to 1.32) p=0.38 | 0.57 (0.16 to 2.10) p=0.40 | 0.49 (0.26 to 0.93) p=0.03 | 1.27 (0.56 to 2.91) p=0.57 |
| - 75+ | 0.36 (0.21 to 0.62) p<0.001 | 0.30 (0.11 to 0.83) p=0.02 | 1.75 (0.54 to 5.69) p=0.35 | 0.32 (0.11 to 0.88) p=0.03 | 0.49 (0.11 to 2.16) p=0.34 |
| Hospital day at entry (N=657) |  |  |  |  |  |
| - 0-3 | Reference | Reference | Reference | Reference | Reference |
| - 4-7 | 0.75 (0.53 to 1.06) p=0.10 | 1.21 (0.69 to 2.14) p=0.51 | 0.82 (0.23 to 2.91) p=0.73 | 0.67 (0.37 to 1.23) p=0.20 | 0.48 (0.21 to 1.12) p=0.09 |
| - 8+ | 0.69 (0.46 to 1.04) p=0.08 | 0.80 (0.39 to 1.63) p=0.54 | 1.56 (0.43 to 5.63) p=0.50 | 0.61 (0.30 to 1.26) p=0.18 | 0.35 (0.11 to 1.11) p=0.08 |
| Gender |  |  |  |  |  |
| - Male | Reference | Reference | Reference | Reference | Reference |
| - Female | 1.38 (1.01 to 1.88) p=0.04 | 0.80 (0.47 to 1.38) p=0.43 | 1.07 (0.37 to 3.09) p=0.90 | 2.66 (1.57 to 4.53) p<0.001 | 0.56 (0.21 to 1.49) p=0.25 |
| Diagnosis^✝^ |  |  |  |  |  |
| - Medical | Reference | Reference | Reference | Reference | Reference |
| - Surgical elect (Not cardiac) | 1.10 (0.65 to 1.85) p=0.73 | 0.75 (0.28 to 1.99) p=0.56 | 1.56 (0.43 to 5.75) p=0.49 | 1.06 (0.41 to 2.78) p=0.90 | 1.72 (0.54 to 5.46) p=0.36 |
| - Surgical Cardiac | 0.73 (0.41 to 1.30) p=0.28 | 0.97 (0.43 to 2.18) p=0.94 | 1.50 (0.41 to 5.48) p=0.54 | 0.57 (0.17 to 1.92) p=0.36 | 0.39 (0.05 to 3.08) p=0.37 |
| - Surgical emergency (Not trauma) | 2.35 (1.59 to 3.47) p<0.001 | 1.55 (0.82 to 2.94) p=0.18 | n/c | 3.18 (1.67 to 6.07) p<0.001 | 3.17 (1.25 to 8.03) p=0.02 |
| - Trauma and burns | 2.19 (1.43 to 3.33) p<0.001 | 2.61 (1.42 to 4.79) p=0.002 | 0.92 (0.19 to 4.58) p=0.92 | 2.24 (1.05 to 4.80) p=0.04 | 0.91 (0.20 to 4.22) p=0.91 |
| ICU patients   - ICU APACHE II (N=662)   - 0-9   - 10-19   - 20-29   - 30-49 | 1.23 (0.73 to 2.07) p=0.43  Reference  0.62 (0.44 to 0.86) p=0.005  0.58 (0.31 to 1.08) p=0.09 | 0.40 (0.13 to 1.30) p=0.13  Reference  0.51 (0.30 to 0.86) p=0.01  0.35 (0.10 to 1.15) p=0.08 | n/c  Reference  1,18 (0.42 to 3.30) p=0.76  1.66 (0.35 to 7.95) p=0.53 | 1.68 (0.78 to 3.65) p=0.19  Reference  0.50 (0.26 to 0.97) p=0.04  0.66 (0.24 to 1.86) p=0.44 | 3.47 (1.18 to 10.20) p=0.02 Reference  1.20 (0.48 to 3.02) p=0.70  1.16 (0.24 to 5.51) p=0.85 |
| Diabetes (N=663)   - No - Yes | Reference  0.79 (0.53 to 1.19) p=0.26 | Reference  1.10 (0.62 to 1.97) p=0.74 | Reference  2.64 (0.96 to 7.28) p=0.06 | Reference  0.15 (0.04 to 0.63) p=0.01 | Reference  1.04 (0.39 to 2.77) p=0.94 |
| Artery – AC   - Radial - Femoral - Dorsalis pedis - Other | Reference  0.23 (0.74 to 2.03) p=0.42  1.46 (0.64 to 3.30) p=0.37  0.49 (0.23 to 1.05) p=0.07 | Reference  2.21 (1.15 to 4.23) p=0.02  0.54 (0.08 to 3.43) p=0.51  0.60 (0.19 to 1.92) p=0.39 | Reference  0.68 (0.08 to 5.65) p=0.72  2.43 (0.29 to 20.07) p=0.41  0.88 (0.13 to 5.80) p=0.90 | Reference  0.21 (0.03 to 1.48) p=0.12  3.03 (1.08 to 8.47) p=0.04  0.22 (0.03 to 1.59) p=0.13 | Reference  1.55 (0.48 to 5.03) p=0.47  n/c  0.53 (0.07 to 3.87) p=0.53 |
| Current infection (at entry)   - No - Yes | Reference  0.95 (0.65 to 1.40) p=0.81 | Reference  1.42 (0.83 to 2.44) p=0.21 | Reference  0.97 (0.28 to 3.39) p=0.96 | Reference  0.74 (0.34 to 1.57) p=0.43 | Reference  0.58 (0.17 to 1.93) p=0.38 |
| Site check (N=662) (Total = 100%) |  |  |  |  |  |
| - Normal | Reference | Reference | Reference | Reference | Reference |
| - Abnormal | 2.09 (1.31 to 3.34) p=0.002 | 2.70 (1.47 to 4.96) p=0.001 | n/c | 1.55 (0.64 to 3.74) p=0.33 | 0.67 (0.09 to 5.07) p=0.70 |
| Multiple insertion attempts (n=653)   - No - Yes | Reference  1.52 (0.62 to 3.71) p=0.36 | Reference  0.62 (0.08 to 4.48) p=0.63 | Reference  n/c | Reference  1.74 (0.40 to 7.60) p=0.46 | Reference  n/c |
| Ultrasound guided insertion (n=657)   - No - Yes | Reference  0.45 (0.30 to 0.69) p<0.001 | Reference  0.56 (0.30 to 1.06) p=0.08 | Reference  1.79 (0.66 to 4.81) p=0.25 | Reference  0.11 (0.03 to 0.47) p=0.003 | Reference  0.62 (0.21 to 1.81) p=0.39 |
| Place of insertion   - ICU - Other | Reference  1.32 (0.97 to 1.78) p=0.08 | Reference  1.29 (0.80 to 2.07) p=0.30 | Reference  0.42 (0.12 to 1.46) p=0.17 | Reference  1.30 (0.76 to 2.22) p=0.33 | Reference  1.66 (0.76 to 3.63) p=0.20 |
| Patient ventilated   - No - Yes | Reference  1.03 (0.71 to 1.49) p=0.88 | Reference  1.05 (0.59 to 1.85) p=0.87 | Reference  4.05 (0.54 to 20.34) p=0.17 | Reference  0.81 (0.43 to 1.51) p=0.50 | Reference  1.03 (0.39 to 2.72) p=0.96 |
| Received IV antibiotics (ever)   - No - Yes | Reference  0.65 (0.45 to 0.93) p=0.02 | Reference  0.58 (0.33 to 1.01) p=0.06 | Reference  n/c | Reference  0.50 (0.28 to 0.90) p=0.02 | Reference 0.78 (0.29 to 2.09) p=0.63 |
| Received IV heparin infusion (ever) |  |  |  |  |  |
| - No - Yes | Reference  0.61 (0.35 to 1.09) p=0.10 | Reference  0.91 (0.41 to 2.04) p=0.82 | Reference  n/c | Reference  0.53 (0.17 to 1.72) p=0.29 | Reference  0.83 (0.19 to 3.60) p=0.81 |
| n/c: not calculable; HR: hazard ratio; SHR: sub hazard ratio; BSI: bloodstream infection; ICU: intensive care unit; APACHE: The Acute Physiology and Chronic Health Evaluation; IV: intravenous.  ^✝^4 observations with other diagnoses were omitted due to absence of any device failures.  ^3 observations with advanced transparent dressings alone were omitted due to absence of any device failures. | | | | | |
