# Supplementary material for: Risk factors for arterial catheter failure and complications during critical care hospitalisation: a secondary analysis of a multisite, randomised trial
Source: J Intensive Care. 2024 Mar 8;12:12. doi: 10.1186/s40560-024-00719-1 (PMC10924392; doi:10.1186/s40560-024-00719-1)
Supplement: Supplementary file 2 — Additional file 2. Bayesian information criterion values by failure type. [file 40560_2024_719_MOESM2_ESM.docx]

**Additional file 2.** **Bayesian information criterion values by failure type**

| **Number of variables** | **BIC** | **Variables** | | | | | | | |
| --- | --- | --- | --- | --- | --- | --- | --- | --- | --- |
| **All cause failure (N=655)** | | | | | | | | | |
| Null model | 1928.4 |  | |  | |  | |  | |
| 1 | 1919.9 | Tech insert | |  | |  | |  | |
| 2 | 1914.1 | Tech insert | | Age | |  | |  | |
| 3 | 1915.7 | Tech insert | | Age | | Site check | |  | |
| 4 | 1918.8 | Tech insert | | Age | | Dressings/ securements | | Gender | |
| **Suspected CABSI (N=655)** | | | | | | | | | |
| Null model | 769.1 |  | |  | |  | |  | |
| 1 | 768.3 | Site check | |  | |  | |  | |
| 2 | 771.6 | Site check | | Tech insert | |  | |  | |
| 3 | 774.5 | Site check | | Tech insert | | IV antibiotics | |  | |
| 4 | 779.3 | Site check | | Tech insert | | IV antibiotics | | Current infection | |
| **Proven CABSI (N=656)** | | | | | | | | | |
| Null model | 171.8 | |  | |  | |  | |  |
| 1 | 175.2 | | Diabetes | |  | |  | |  |
| 2 | 178.7 | | Diabetes | | Patient ventilated | |  | |  |
| 3 | 183.2 | | Diabetes | | Patient ventilated | | Inserted in ICU | |  |
| 4 | 189.1 | | Diabetes | | Patient ventilated | | Inserted in ICU | | Tech insert |
| **Occlusion (N=656)** | | | | | | | | | |
| Null model | 644.4 |  | |  | |  | |  | |
| 1 | 632.8 | Tech insert | |  | |  | |  | |
| 2 | 627.9 | Tech insert | | Diabetes | |  | |  | |
| 3 | 624.3 | Tech insert | | Diabetes | | Gender | |  | |
| 4 | 628.91 | Tech insert | | Gender | | Diabetes | | IV Antibiotics (ever) | |
| **Accidental removal (N=657)** | | | | | | | | | |
| Null model | 294.5 |  | |  | |  | |  | |
| 1 | 299.8 | Gender | |  | |  | |  | |
| 2 | 305.3 | Gender | | Place of insertion | |  | |  | |
| 3 | 311.1 | Gender | | Place of insertion | | Tech insert | |  | |
| 4 | 316.8 | Gender | | Place of insertion | | Tech insert | | Current infection | |
| BIC: Bayesian information criterion; Tech: technology assisted insertion (ultrasound); IV: intravenous | | | | | | | | | |
